# Ancient Chinese Formula Qiong-Yu-Gao Protects Against Cisplatin-Induced Nephrotoxicity Without Reducing Anti-tumor Activity

**DOI:** 10.1038/srep15592

**Published:** 2015-10-29

**Authors:** Zhi-Ying Teng, Xiao-Lan Cheng, Xue-Ting Cai, Yang Yang, Xiao-Yan Sun, Jin-Di Xu, Wu-Guang Lu, Jiao Chen, Chun-Ping Hu, Qian Zhou, Xiao-Ning Wang, Song-Lin Li, Peng Cao

**Affiliations:** 1 Jiangsu Key Laboratory for Pharmacology and Safety Evaluation of Chinese Materia Medica, Affiliated Hospital of Integrated Traditional Chinese and Western Medicine, Nanjing University of Chinese Medicine, Nanjing 210023, China; 2 Laboratory of Cellular and Molecular Biology, Jiangsu Province Academy of Traditional Chinese Medicine, Nanjing 210028, China; 3 Department of Pharmaceutical Analysis and Metabolomics, Jiangsu Province Academy of Traditional Chinese Medicine, Nanjing 210028, PR China

## Abstract

Cisplatin is a highly effective anti-cancer chemotherapeutic agent; however, its clinical use is severely limited by serious side effects, of which nephrotoxicity is the most important. In this study, we investigated whether Qiong-Yu-Gao (QYG), a popular traditional Chinese medicinal formula described 840 years ago, exhibits protective effects against cisplatin-induced renal toxicity. Using a mouse model of cisplatin-induced renal dysfunction, we observed that pretreatment with QYG attenuated cisplatin-induced elevations in blood urea nitrogen and creatinine levels, ameliorated renal tubular lesions, reduced apoptosis, and accelerated tubular cell regeneration. Cisplatin-mediated elevations in tumor necrosis factor alpha (TNF-α) mRNA, interleukin-1 beta (IL-1β) mRNA, and cyclooxygenase-2 (COX-2) protein in the kidney were also significantly suppressed by QYG treatment. Furthermore, QYG reduced platinum accumulation in the kidney by decreasing the expression of copper transporter 1 and organic cation transporter 2. An *in vivo* study using implanted Lewis lung cancer cells revealed that concurrent administration of QYG and cisplatin did not alter the anti-tumor activity of cisplatin. Our findings suggest that the traditional Chinese medicinal formula QYG inhibits cisplatin toxicity by several mechanisms that act simultaneously, without compromising its therapeutic efficacy. Therefore, QYG may be useful in the clinic as a protective agent to prevent cisplatin-induced nephrotoxicity.

Cisplatin is a first-line chemotherapeutic drug that is widely used to treat patients with several types of solid tumors, including lung, head and neck, bladder, cervical, ovarian, endometrial, and testicular cancers^1^,^2^. However, cisplatin therapy is often restricted by its serious adverse effects, particularly damage to the kidneys (nephrotoxicity)^3^,^4^. Approximately 20–30% of patients treated with cisplatin experience a reversible decline in renal function after the first course of therapy^5^. Despite being the focus of intense investigation in recent years, the mechanisms underlying cisplatin-related nephrotoxicity are not understood in detail^6^.

It has been suggested that renal cell apoptosis, inflammation, necrosis, and oxidative stress are likely to contribute to cisplatin-induced nephrotoxicity; however, the initiating event and detailed mechanisms underlying tubular damage are poorly understood^7^,^8^. Recently, considerable attention has been paid to the roles of specific cellular uptake processes in cisplatin toxicity. The copper transporter 1 (CTR1) and organic cation transporter 2 (OCT2), which are involved in cisplatin accumulation in the kidney, have been proposed as potential targets for protective therapeutic interventions that could accompany cisplatin treatment and decrease its toxicity^9^,^10^.

At present, several therapeutic strategies, including intensive hydration and the use of alternative cisplatin analogs, have been proposed as possible approaches for preventing cisplatin-induced nephrotoxicity. However, clinical outcomes using such treatments have been unsatisfactory; hydration therapy did not resolve renal dysfunction in a significant percentage of treated patients, whereas cisplatin analogs were not as effective as cisplatin^11^,^12^,^13^. Discontinuation of cisplatin therapy remains the only option for patients with progressive renal failure. Therefore, identifying effective methods for preventing cisplatin-induced renal injury is a critical issue in cancer therapeutic research.

Herbs and plant-based formulations have been used by practitioners of traditional Chinese medicine (TCM) to treat human diseases and reduce the effects of toxic substances for millennia. Qiong-Yu-Gao (QYG) is a classical tonic TCM formula that was first mentioned in the “Hong-Shi-Ji-Yan-Fang,” written in 1170. QYG consists of Rehmanniae Radix (RR), Poriae (PO), and Ginseng Radix (GR) in a weight ratio of 7:2:1 and is used to invigorate the spleen, strengthen the kidney, nourish the yin, and replenish qi (Fig. 1a)^14^. In a previous work, we reported a comprehensive chemical investigation of QYG^15^. We revealed that the principal bioactive compounds of QYG were ginsenosides, phenethylalcohol glycosides, iridoid glycosides, and triterpenoid acids. In addition, an efficient and reliable approach based on UHPLC-PDA-QTOF-MS/MS and chemical profiling was developed for evaluating the quality of QYG.

Among the component herbs of QYG, RR and PO are folk medicines that have been widely used in Asian countries to treat renal diseases^16^,^17^. In modern medical practice, QYG is often used to alleviate the side effects of chemotherapy, including fatigue, nausea, vomiting, anorexia, and bone marrow inhibition^18^. Modern pharmacological studies show that QYG exhibits a wide range of pharmacological effects, including anti-aging, anti-inflammatory, anti-apoptotic, and anti-cancer activities, and protects the gastric mucosa from damage^19^,^20^,^21^. Recently, the renoprotective effects of RR, PO, and GR have been demonstrated in several renal failure models, including cisplatin-induced nephropathy in rodents^22^,^23^,^24^,^25^,^26^,^27^. Moreover, the primary phytochemical compounds isolated from RR, PO, and GR, including catalpol and ginsenosides, significantly improve renal function and prevent pathological changes^28^,^29^.

On the basis of the demonstrated protective benefits of the component herbs comprising QYG and their active compounds against renal injury, we hypothesized that QYG might protect rodents from cisplatin-mediated nephrotoxicity. To test this hypothesis, we evaluated the renoprotective efficacy of QYG against cisplatin-induced kidney injury in mice. Potential molecular mechanisms for the therapeutic effects of QYG were also investigated, including effects on anti-inflammatory and anti-apoptotic signaling, the regenerative capability of the kidneys, and renal cellular transport.

## Methods

### Chemicals and reagents

Cisplatin was purchased from Jiangsu Hospital of Integrated Traditional Chinese and Western Medicine. Primary antibodies against CTR1, OCT2, proliferating cell nuclear antigen (PCNA), cyclooxygenase-2 (COX-2), cleaved poly(ADP-ribose) polymerase 1 (PARP), and cleaved caspase-3 were obtained from Santa Cruz Biotechnology (Santa Cruz, CA, USA), whereas primary antibodies against p53 were obtained from Bioword Technology Inc. (Nanjing, China). For the liquid chromatography-mass spectrometry (LC-MS) analysis, 13 reference compounds were purchased from Sichuan Victory Co. Ltd. (Chengdu, China). High-performance liquid chromatography (HPLC)-MS grade acetonitrile was purchased from TEDIA Company Inc. (Fairfield, OH, USA). Formic acid was purchased from ROE Scientific Inc. (USA). All other solvents and chemicals were of analytical grade. Ultra-pure water was prepared using a Milli-Q SP system (Millipore, Bedford, MA, USA).

### Sample preparation

GR, the root and rhizome of *Panax ginseng*, was collected from professional herbal growers in Jilin province in September 2013. RR (the root of *Rehmannia glutinosa*) and PO (the sclerotium of *Poria cocos*) were supplied by Jiangsu Hospital of Integrated Traditional Chinese and Western Medicine in January 2014. GR, RR, and PO were authenticated by Professor Song-Lin Li according to the monographs documented in the Chinese Pharmacopeia (Part I, 2010 Version). Voucher specimens of GR, RR, and PO (no. JSPACM-03–65-1 for GR; no. JSPACM-02–14 for RR; no. JSPACM-05-01 for PO) were deposited at the Department of Pharmaceutical Analysis and Metabolomics at Jiangsu Province Academy of Traditional Chinese Medicine (Nanjing, China). To prepare QYG samples, RR, PO, and GR were mixed (weight ratio of 7:2:1) and refluxed with 8 volumes of water for 4 h after maceration for 30 min. The solution was filtered and the residue was refluxed with 8 volumes of water for 3 h. The combined filtrations were rotary evaporated at 70 °C to produce a syrupy QYG solution with a density of 1.2–1.6 kg/L. The QYG solution was stored at –80 °C.

Before the LC-MS analysis, the QYG samples were subjected to an ultrasonic extraction in 2 volumes of methanol for 30 min. The temperature of the ultrasonic bath was kept at 25 ± 1 °C with running water. The extracts were filtered through a 0.2 μm PTFE syringe filter and subjected to LC-MS analysis (2.0 μL of each sample).

### LC-MS analysis

LC-MS analysis was performed on a Waters ACQUITY UPLC^TM^ system (Waters Co., Milford, MA, USA). Chromatographic separation was carried out at 35 °C on a Waters ACQUITY HSS T3 column (100 mm × 2.1 mm, 1.8 μm) using the following gradient profile: 1% B (0–0.5 min), 1%–12% B (0.5–3.5 min), 12%–40% B (3.5–15 min), 40%–45% B (15–20 min), 45%–60% B (20–22 min). The mobile phase was composed of 0.1% formic acid (A) and 100% acetonitrile (B). The injection volume was 10 μL.

Detections were performed using a Q-TOF Synapt G2 mass spectrometer (Waters MS Technologies, Manchester, UK) in negative mode. The operating parameters of MS were set up according to our previous study^15^. The accurate mass and elemental composition for the precursor ions and fragment ions were analyzed by the MassLynx V4.1 software (Waters Co., Mil-ford, USA).

Quantification analysis was carried out on a Micromass Quattro-Micro™ triple-quadrupole mass spectrometer (Waters Corp., MA, USA) using the same methods described in our previous work^30^. The concentrations of major constituents were determined using an external standard curve.

### Animals

Male C57BL/6 mice (20–24 g, 8 weeks of age) were obtained from the Institute of Laboratory Animal Science at the Chinese Academy of Medical Sciences (Beijing, China). All mice were fed a standard commercial diet while housed in a light- and temperature-controlled room (21–24 °C, 40–55% humidity). All of the experimental protocols were approved by the Animal Care and Use Committee of Jiangsu Academy of Traditional Chinese Medicine and written following the ARRIVE guidelines. All experiments were performed in accordance with published National Institutes of Health guidelines.

### *In vivo* mouse model of cisplatin-induced nephrotoxicity

According to published methods, nephrotoxicity was induced by intraperitoneal administration of 20 mg/kg cisplatin^5^. The experimental mice were randomly assigned to 3 groups (n = 6): control, cisplatin-treated, and QYG+cisplatin-treated groups. The QYG+cisplatin group was treated orally with 14 g/kg QYG once daily before cisplatin administration for 7 days, followed by oral administration of 14 g/kg QYG once daily after cisplatin administration for 3 days. Both cisplatin and QYG+ cisplatin groups received a single intraperitoneal injection of cisplatin on the 7th day of the experiment. The normal control group was treated orally with saline instead of QYG and/or cisplatin. Body weight was recorded daily. All animals were sacrificed 3 days after cisplatin administration. Urine, blood, and kidneys were collected for assays.

### Renal function detection

For the renal function analysis, serum blood urea nitrogen (BUN) and creatinine (Cr) were measured at day 3 after the end of cisplatin administration using commercial kits supplied by Nanjing Jiancheng Bioengineering Institute (Nanjing, China). Body weight loss and kidney-to-body weight ratios were calculated as indexes of kidney hypertrophy.

### Histological analysis and neutrophil counting

Kidney tissues were fixed and subjected to hematoxylin and eosin (HE) and periodic acid-Schiff (PAS) staining. Morphology was analyzed using a light microscope (Zeiss Axio Observer A1). Tubular damage in HE-stained kidney sections was evaluated and scored based on the percentage of cortical tubular necrosis: 0 = no damage, 1 = 0–10%, 2 = 11%–25%, 3 = 26%–45%, 4 = 46%–75%, 5 = more than 75%^30^. Slides were scored in a blinded manner. Mean scores were calculated by counting 10 different fields for each group.

### Quantitative real-time PCR

Total RNA was isolated from renal tissue and detected by quantitative real-time PCR. Mouse *Gapdh* was used as endogenous control for sample normalization. Results are presented as fold-increases relative to the expression of mouse *Gapdh*. The PCR primer pairs were as follows: mouse *Gapdh*, 5′-TTG CAG TGG CAA AGT GGA GA-3′ (forward) and 5′-GGT CTC GCT CCT GGA AGA TG-3′ (reverse); mouse TNF-α, 5′-GAC CCC TTT ACT CTG ACC CC-3′ (forward) and 5′-AGG CTC CAG TGA ATT CGG AA-3′ (reverse); mouse IL-1β, 5′-ACT CAT TGT GGC TGT GGA GA-3′ (forward) and 5′-TTG TTC ATC TCG GAG CCT GT-3′ (reverse).

### ELISA assays

To quantify TNF-α and IL-1β protein levels, 50 mg of renal tissue was immersed in 450 mL of PBS and ground into a powder in liquid nitrogen. The extracts were centrifuged at 3000 rpm for 10 min at 4 °C to remove insoluble material. After total protein content determination, the supernatant fractions were analyzed using ELISA kits (Shanghai Meilian Science & Technology Co., Ltd, Shanghai, China) according to the manufacturer’s instructions. After the enzyme-substrate reaction, the absorbance of each sample was measured at 450 nm with a microplate reader. Standard curves were prepared using diluted standard solutions to allow calculation of the TNF-α and IL-1β protein levels in the samples. All standards and samples were run in duplicate.

### TUNEL staining and PCNA assay

Cellular apoptosis in renal tissue was assessed using a terminal deoxynucleotidyl transferase dUTP nick end-labeling (TUNEL) assay kit (Beyotime, China). Briefly, following dewaxing and hydration, kidney sections were digested with proteinase K and labeled with a TUNEL reaction mixture for 60 min at 37 °C. To determine the number of proliferating tubular cells, expression of PCNA was detected by immunohistochemistry according to a previously published method^31^.

### Immunoblotting assay

The kidneys were lysed in RIPA buffer and centrifuged at 12,000 rpm for 15 min at 4 °C. The supernatants containing total protein were analyzed by 12% SDS-PAGE and transferred onto PVDF membranes. After blocking, the membranes were incubated overnight with primary antibodies against p53. Next, the membranes were incubated with secondary antibodies and detected with an enhanced chemiluminescence detection kit (GE Healthcare Bio-Sciences, Pittsburgh, PA, USA). The intensity of each band was determined using ImageJ software (National Institutes of Health, Bethesda, MD, USA).

### Immunohistochemical analysis

For immunohistochemistry, 4-μm thick deparaffinized tissue sections were rehydrated with a series of xylene and aqueous alcohol solutions. The deparaffinized renal slices were incubated overnight at 4 °C with primary antibodies against CTR1, OCT2, COX-2, cleaved PARP, and cleaved caspase 3. Next, the slices were incubated with biotin-labeled secondary antibodies and streptavidin-HRP for 30 min at room temperature. Immunoreactivity was detected using 3–3-diaminobenzidine (DAB), followed by counterstaining with hematoxylin. Localization of positive staining was analyzed by light microscopy.

### Determination of platinum in the kidney and urine

Platinum content in the kidney and urine was determined by inductively coupled plasma spectrometry (ICP-MS) according to the method described by Verbanac *et al.*^32^.

### Xenograft tumor model in mice

Lewis lung cancer cell xenograft tumors were established in C57BL/6 mice as previously reported^33^. When the tumors had grown to 200–300 mm^3^, the mice were randomly assigned to 4 groups (n = 13 each). The cisplatin group was intraperitoneally injected with 2 mg/kg cisplatin once every other day, for a total of 7 doses. The group treated with cisplatin and QYG received cisplatin (2 mg/kg, i.p.) once every other day and QYG (14 g/kg, i.g.) once daily for 13 days. The group treated with QYG only received 13 doses of QYG (14 g/kg, i.g.). The positive control group (tumor model group) was not treated with QYG or cisplatin. Tumor size was measured every day. At the end of the treatment period, the mice were sacrificed and tumors were collected.

### Statistical analysis

All values are presented as mean ± SD. Student’s *t*-test was used to analyze the differences between the groups. Differences were considered statistically significance at *P < *0.05.

## Results

### Phytochemical analysis of QYG by LC-MS

**Figure 1:**
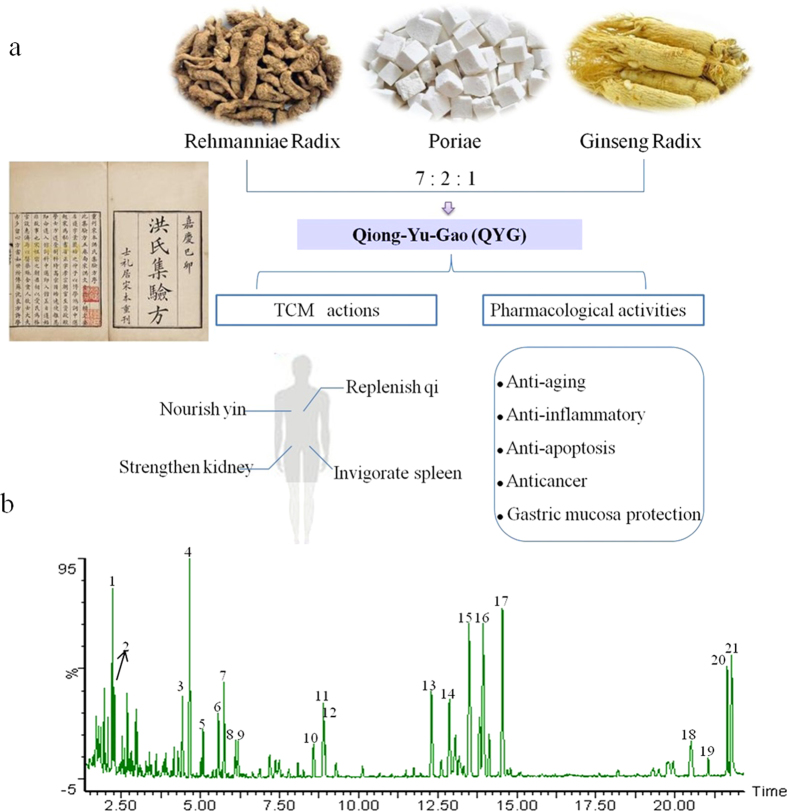
QYG recorded in Hong-Shi-Ji-Yan-Fang and its pharmacological activities (a).Representative base peak intensity (BPI) chromatograms of QYG analyzed by LC-MS (**b**). Figure 1a was taken by Dr. Xiao-Lan Cheng, one of the authors of this work. Figure 1b was resulted from LC-MS analysis of QYG performed by author Jin-Di Xu.

LC-MS analysis was conducted on QYG extract to confirm its biological composition, resulting in the identification of 21 major components, of which 13 were confirmed by using reference compounds. The remaining 8 constituents were tentatively assigned identities by matching the empirical molecular formula of each constituent with that of a known compound and/or elucidating quasi-molecular ions and fragment ions. A typical base peak intensity (BPI) chromatogram of a QYG sample is presented in Fig. 1b. The structures of all identified compounds are presented in Supplementary Figure S1. The properties of the identified compounds are summarized in Table 1. In order to evaluate the quality of the samples, an external standard method was applied to quantitatively analyze 13 major compounds in the QYG samples. The external standard method was validated in terms of linearity, precision, accuracy, and stability. The quantitative results are presented in Table 1.

### QYG inhibits cisplatin-induced acute kidney injury in mice

**Figure 2:**
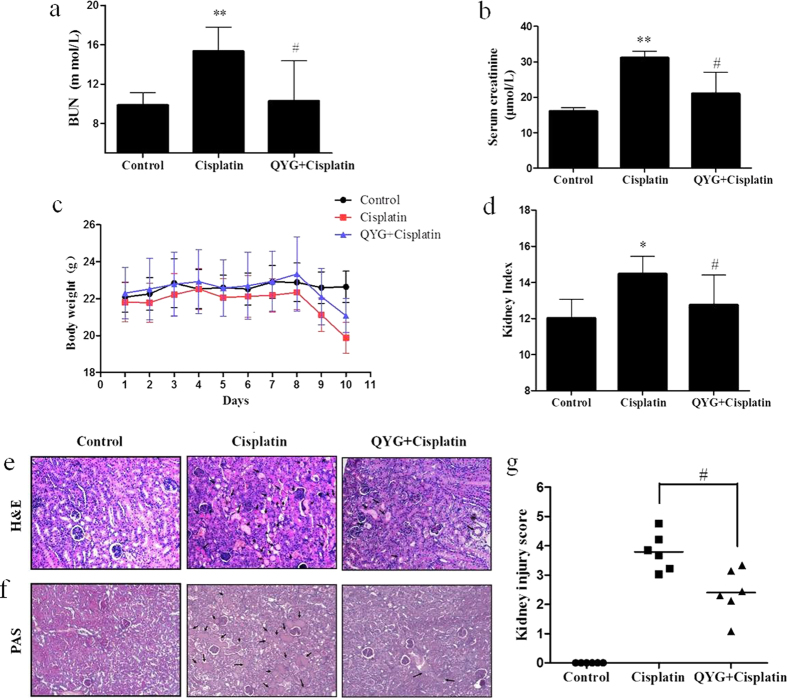
Effects of QYG on BUN (**a**) and Cre (**b**) levels in plasma, body weight (c), and kidney-to-body weight ratio (d) in cisplatin-treated mice. Renal tissue obtained from treated mice was stained with hematoxylin and eosin (H&E, (**e**)) and periodic acid-Schiff stain (PAS, (**f**)). The severity of tubular injury was quantified by observing stained tissue samples under a light microscope. Tubular injury (**g**) was scored using the quantitative evaluation method, as described in the Methods section. Damaged areas of the tissue sections are marked with black arrows (200 × magnification). Control, saline-treated control group; cisplatin, animals administered 20 mg/kg cisplatin alone; cisplatin+QYG, animals administered cisplatin and 14 g/kg QYG. All data are expressed as mean ± SD (n = 6). *P < 0.05; **P < 0.01, in comparison with the normal control group; ^#^*P *< 0.05, in comparison with the group treated with cisplatin alone.

Cisplatin-induced renal damage was evaluated by measuring biochemical markers, including BUN level, Cre level, and ratio of kidney weight to body weight. Compared with normal control group, serum BUN and Cre levels were observed to be significantly higher in the cisplatin-treated group at day 3 after cisplatin administration (*P *= 1.84 × 10^−8^ and 0.0413, respectively, Fig. 2a,b). The serum BUN and Cre levels of the group pretreated with 14 g/kg QYG before cisplatin administration were significantly lower than those of the group treated with cisplatin alone (*P *= 0.0050 and 0.0494, respectively). Additionally, reduced body weight and an increased ratio of kidney weight to body weight were recorded in the group treated with cisplatin alone, providing evidence of renal injury. QYG treatment attenuated cisplatin-induced increase in kidney to body weight ratios and decrease in body weight (Fig. 2c,d).

### QYG ameliorates cisplatin-induced renal tubular damage in mice

The pathophysiology of cisplatin-induced renal injury can be classified into 4 types of toxicity: tubular toxicity, vascular damage, glomerular injury, and interstitial injury. We examined cisplatin-induced tubular damage and the potential protective effect of QYG by staining kidney specimens with HE and PAS stains. As shown in Fig. 2e, the animals in the control group exhibited normal renal tissue architecture. Cisplatin treatment elicited obvious signs of tubular damage, including tubular degeneration, swelling, vacuole formation, and necrosis, which were markedly attenuated by pretreatment with QYG. Consistent with the findings from the HE staining experiments, deleterious structural changes, including brush border membrane loss, PAS-positive material deposition, and cast formation were observed in renal tissue samples from cisplatin-treated mice. Co-administration of QYG with cisplatin suppressed the deleterious structural changes induced by cisplatin (Fig. 2f). To compare the tubular damage caused by cisplatin in each treatment group in a quantitative manner, the extent of injury was scored as the percentage of cortical tubular necrosis. As shown in Fig. 2g, cisplatin significantly increased the extent of tubular injury (3.79 ± 0.636) compared with control animals, whereas QYG significantly decreased the injury score (2.40 ± 0.806, P = 0.042), suggesting that QYG could be used to prevent cisplatin-induced tubular injury.

### QYG inhibits potential mediators of cisplatin-induced renal damage in mice

**Figure 3:**
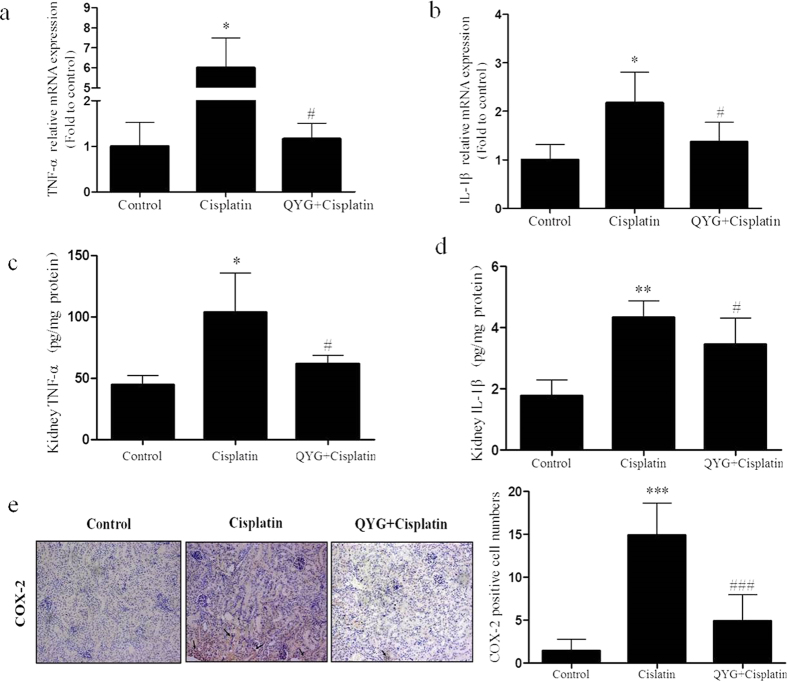
Effect of QYG on cisplatin-induced proinflammatory mediators of renal damage. mRNA and protein levels of TNF-α (*Tnf*; (**a**,**c**)) and IL-1β (*Il1b*; (**b**,**d**)) in renal tissue samples were analyzed by RT-PCR and ELISA, respectively, while COX-2 expression (**c**) was evaluated using an immunohistochemistry assay. *P < 0.05, **P <  0.01, in comparison with the control group; ^#^*P *< 0.05, ^###^*P* <  0.001 in comparison with the group administered cisplatin alone.

Inflammatory cytokines such as TNF-α, IL-1β, and COX-2 may contribute to the induction and pathological progression of cisplatin-induced nephrotoxicity^34^. In order to investigate the effects of QYG treatment on the inflammatory response in experimental mice, we measured mRNA expression levels of TNF-α, IL-1β, and COX-2 in renal tissue. Animals treated with cisplatin had significantly higher TNF-α and IL-1β mRNA levels (Fig. 3a,b, *P *= 0.0113 and 0.0155, respectively) than the corresponding levels in the control group. Pretreatment with QYG significantly decreased TNF-α (*P *= 0.0223) and IL-1β (*P *= 0.0168) mRNA levels compared with cisplatin group. The ELISA assays further confirmed the RT-PCR results by demonstrating increased protein levels of TNF-α and IL-1β in cisplatin-treated mice, which were reduced in mice pretreated with QYG before cisplatin administration, indicating that modulation of cisplatin-induced inflammatory cytokine expression by QYG occurred at the protein and mRNA levels (Fig. 3c,d). Similarly, immunohistochemistry assays demonstrated that COX-2 expression in the kidney also increased because of cisplatin treatment, whereas co-administration of QYG with cisplatin remarkerly attenuated this effect (Fig. 3e).

### QYG reduces apoptosis and accelerates tubular cell regeneration in cisplatin-treated mice

**Figure 4:**
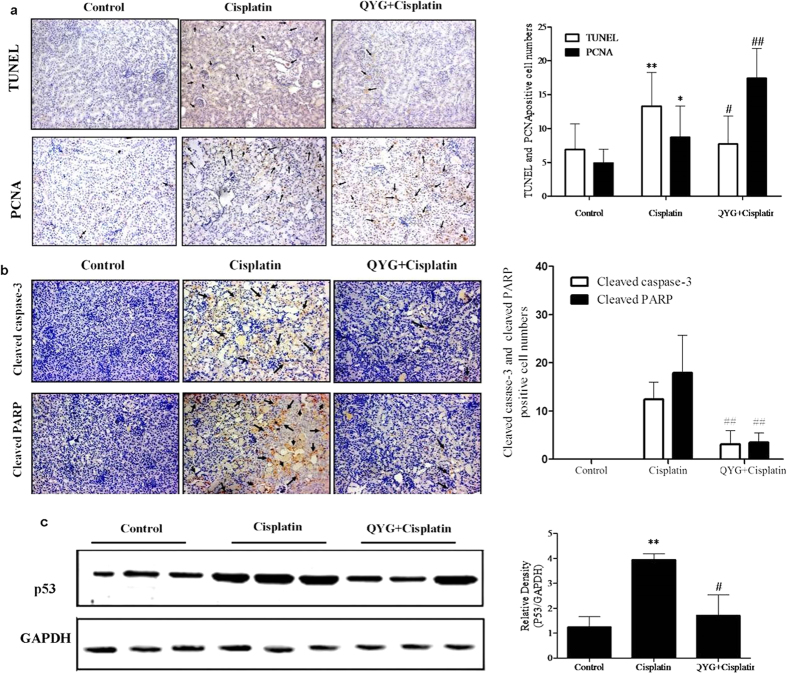
Effects of QYG on cisplatin-induced tubular apoptosis and tubular cell proliferation 3 days after cisplatin administration. The animals were treated as indicated, after which fixed kidney tissue samples were analyzed by TUNEL staining and PCNA staining. (**a**) Representative images of TUNEL- and PCNA-stained kidney sections. (**b**) Expression of cleaved caspase-3 and cleaved PARP in the kidney was evaluated using immunohistochemistry. (**c**) Total protein expression of p53 in cisplatin-treated mice was evaluated by immunoblot analysis (n = 3 per lane). GAPDH was used as a loading control. *P < 0.05; **P < 0.01, in comparison with the control group; ^#^*P *< 0.05; ^##^*P *< 0.01, in comparison with the cisplatin group.

Apoptosis of renal tubular cells contributes to cisplatin-mediated acute kidney injury^35^. TUNEL staining was performed to evaluate the effect of QYG on cisplatin-induced apoptosis (Fig. 4a,b). Very few tubular epithelial cells were stained positive in the TUNEL assay performed on the kidneys of the normal control group. Conversely, the cisplatin-treated group had a markedly higher number of TUNEL-positive cells than did the control group (P < 0.01). Mice pretreated with QYG exhibited significantly lower number of TUNEL-positive cells (P=0.0160, compared to cisplatin-treated group). To confirm the apoptosis signaling pathways regulated by QYG, investigation of the apoptosis related proteins was carried out next. As shown in Fig. 4b, c, the group treated with cisplatin showed increased protein expression of p53, cleaved PARP, and caspase-3 in renal tissue in comparison with the corresponding protein levels in the control group. However, pretreatment with QYG before cisplatin administration significantly inhibited cisplatin-induced up-regulation of p53, cleaved PARP, and caspase-3 protein levels. Further evidence of the protective effect of QYG against kidney injury was provided the examination of tubular cell regeneration by using PCNA staining, which showed a dramatically increased number of PCNA-positive cells in animals pretreated with QYG before cisplatin treatment compared with mice treated with cisplatin alone (P = 0.0172, Fig. 4a). These results indicate that QYG promotes renal regeneration and exerts an anti-apoptotic effect that inhibits cisplatin-induced nephrotoxicity.

### QYG decreased platinum accumulation and enhanced platinum efflux

**Figure 5:**
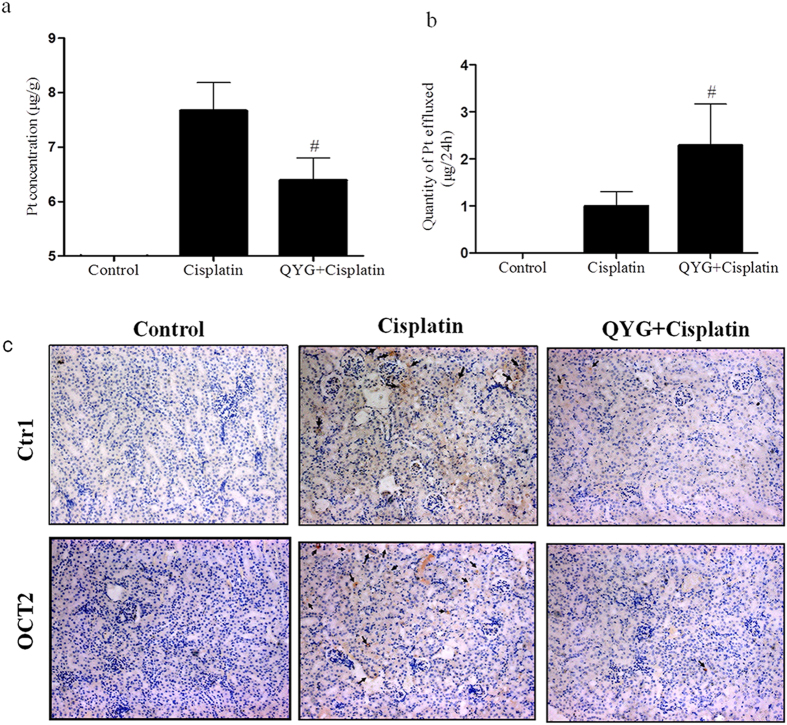
Effects of QYG on platinum accumulation in the kidney and urinary platinum excretion in mice with cisplatin-induced kidney injury. (**a**) Inductively coupled plasma mass spectrometry (ICP-MS) was used to measure platinum concentration in kidney tissue samples. (**b**) Total quantity of platinum effluxed through the urine over 24 hours, measured immediately before the animals were sacrificed. (**c**) Expression of copper transporter 1 (CTR1) and organic anion transporter 2 (OTC2) were quantified by immunohistochemistry assay (200 × magnification). Data are expressed as mean ± SD (n = 5). ^#^*P *< 0.05, in comparison with the group treated with cisplatin alone.

The protective effect of QYG against cisplatin-induced kidney injury could reflect its tendency to reduce platinum accumulation in the kidney, protecting it from cellular damage. To confirm this hypothesis, we measured levels of accumulated platinum in the kidney and urine of cisplatin-treated animals after acid mineralization using ICP-MS. As expected, cisplatin administration resulted in a high concentration of platinum in the kidneys (7.66 ± 0.522 μg/g, Fig. 5a). Treatment with QYG markedly reduced kidney platinum levels compared with animals treated with cisplatin alone (6.39 ± 0.408 μg/g, *P *= 0.0102). To explore the effect of QYG on cisplatin efflux, urine was collected during the 24 h before the animals were sacrificed. As shown in Fig. 5b, acute cisplatin intoxication resulted in decreased platinum efflux (0.99  ± 0.310 μg), whereas treatment with QYG significantly increased the platinum concentration in urine (2.28 ± 0.881 μg, *P *= 0.0476). The decreased renal platinum accumulation and enhanced platinum efflux observed in the animals pretreated with QYG were accompanied by reduced expression levels of CTR1 and OCT2, which are implicated in the cellular uptake of cisplatin (Fig. 5c). These findings provide further evidence of the nephroprotective effects of QYG against cisplatin toxicity.

### QYG does not attenuate the *in vivo* anti-tumor activity of cisplatin

**Figure 6:**
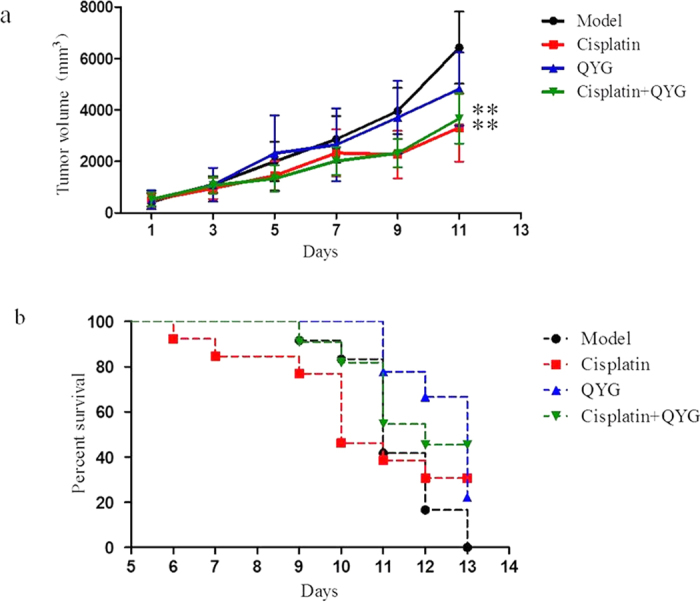
Effect of QYG on the anti-tumor activity of cisplatin against Lewis lung cancer xenograft tumors in C57BL/6 mice. C57BL/6 mice were transplanted with Lewis lung cancer cells and treated as shown. (**a**) Tumor size was measured throughout the experiment and (**b**) survival of tumor-bearing mice was recorded. Data are expressed as mean ± SD (n = 13). In comparison with the model control group, the QYG, cisplatin, and cisplatin + QYG groups showed reduced tumor size (P = 0.006, 0.095, and 0.008, respectively). **P < 0.01, in comparison with the control group.

The effect of QYG on the anti-tumor activity of cisplatin was evaluated using a Lewis lung cancer xenograft model. Oral administration of 14 g/kg QYG for 13 days inhibited tumor growth by 25%, whereas i.p. cisplatin administration inhibited tumor growth by 48% (Fig. 6a). Animals pretreated with QYG before cisplatin administration exhibited a tumor growth inhibition rate of 43% (*P *= 0.627, in comparison with the rate recorded in animals treated with cisplatin alone), suggesting that QYG did not significantly alter the anti-tumor activity of cisplatin. Furthermore, pretreatment with QYG significantly increased the survival of tumor-bearing mice that were treated with cisplatin. The survival rates (number of living mice 13 days after cisplatin treatment) of the groups treated with QYG, cisplatin, and a combination of the 2 agents were 23.07%, 30.77%, and 46.15%, respectively (Fig. 6b). As for survival time, the median survival time of mice in control group was 11.33 ± 1.15 days, whereas in cisplatin- and cisplatin+QYG-treated groups these were 12.23 ± 4.85 and 14.38 ± 3.64 days, respectively. The median survival time of the group treated with QYG alone (14 mg/kg) was 13.55 ± 2.83 days. The median survival time of the cisplatin-treated group was longer than that of the control group, but this difference was not statistically significant (*P *= 0.521). QYG pretreatment before cisplatin injection could significantly extend the survival time of tumor-bearing mice (*P *= 0.008, compared to the model control group). However, the median survival times of the cisplatin + QYG and cisplatin-treated groups were not significantly different (*P *= 0.213). These preliminary results suggest that QYG can prevent cisplatin-induced kidney toxicity without compromising its anti-tumor activity.

## Discussion

Nephrotoxicity is the most significant and dose-limiting side effect encountered in cisplatin-based chemotherapy, placing a considerable health and economic burden on patients. No effective nephroprotective agents are available to counter cisplatin-induced renal toxicity. Although many renoprotective approaches are currently being evaluated, the protective effects of such agents are limited, providing the rationale for developing combined strategies. In the present study, we report for the first time that systemic administration of QYG, an ancient TCM formula, significantly suppresses cisplatin-induced renal injury in mice by attenuating renal inflammation, apoptosis, and platinum accumulation.

Cisplatin-induced renal injury is caused by accumulated cisplatin in the tubules^36^. In the present study, biomarkers of cisplatin-induced renal injury included significantly increased serum BUN and Cre levels, increased kidney relative weight, and elevated kidney-to-body weight ratio; however, pretreatment with QYG (14 g/kg) attenuated these nephrotoxic changes (Fig. 2). Using histopathological analyses, we detected degeneration of tubular structures, with vacuolization and loss of architecture, in cisplatin-treated animals. Conversely, animals treated with QYG for 10 days showed normal kidney morphology. Therefore, QYG appears to be a nephroprotective agent that could be used in the clinic to prevent cisplatin-induced renal damage.

Inflammatory cytokines play an important role in the pathogenesis of renal damage^37^. Cisplatin activates the nuclear factor kappa B (NF-κB) pathway, inducing expression of several proinflammatory genes, including TNF-α, IL-1β, and COX-2 37,38. Suppression of NF-κB activation and inhibition of the secretion of inflammatory cytokines reduces cisplatin nephrotoxicity^39^. In our study, cisplatin treatment significantly increased renal expression of TNF-α, IL-1β, and COX-2. Administration of 14 g/kg QYG attenuated cisplatin-induced overexpression of TNF-α, IL-1β, and COX-2 (Fig. 3). These findings are in agreement with those of previous reports showing that the renoprotective activity of TCM agents (berberine, curcumin, and *Ginkgo biloba* extract) is usually accompanied by inhibition of NF-κB activation and reduced proinflammatory cytokine expression^40^,^41^,^42^.

Tubular cell apoptosis is a characteristic feature of cisplatin nephrotoxicity that results in the loss of renal endothelial cells and renal dysfunction. After cisplatin administration, p53 is rapidly up-regulated and induces apoptosis in tubular cells^43^. In our current study, the number of TUNEL-positive cells in the kidneys of the cisplatin-treated mice was much higher than that of the control mice, whereas pretreatment with QYG significantly reduced the number of TUNEL-positive cells. The decreased protein levels of cleaved PARP, cleaved caspase-3, and p53 confirmed the anti-apoptotic properties of QYG. PCNA expression provides an index of renal regeneration. Attenuation of cisplatin-induced acute renal failure is associated with augmented PCNA expression^44^. The increased number of PCNA-positive cells observed in the QYG-treated mice suggests that QYG promotes tubular cell proliferation, while inhibiting apoptosis. These observations indicate that the renoprotective effects of QYG may be mediated by its anti-apoptotic function.

OCT2, a kidney-specific organic cation transporter, is involved in the uptake of cationic substances such as cisplatin from the circulation into tubular cells. Growing evidence suggests that cisplatin-induced kidney injury is dependent on OCT2-mediated renal tubular transport^45^. OCT2 deletion or inhibition results in significantly impaired uptake and urinary excretion of cisplatin, providing a further rationale for targeting OCT2 for protective interventions^46^. In addition to OCT2, CTR1 was also found to contribute to cisplatin uptake during the pathological progression of cisplatin nephrotoxicity^47^. While CTR1 is expressed at high levels in renal tubular cells, its role in cisplatin-induced nephrotoxicity is unknown. Consistent with previous reports, the results of our current study showed lower platinum content in kidney tissue accompanied by decreased OCT2 and CTR1 expression in QYG-treated mice. These findings suggest that QYG treatment suppresses OCT2 and CTR1 expression, which may contribute to the protective effect of QYG against cisplatin-induced renal injury.

Identification of novel renoprotective strategies that do not diminish the anti-cancer efficacy of cisplatin is essential for the development of clinically applicable interventions. In this study, we demonstrated the protective effect of QYG against cisplatin-induced kidney injury. In order to be an effective therapeutic agent, QYG should not reduce the tumoricidal effect of cisplatin. Therefore, tumor-bearing C57BL/6 mice were subcutaneously injected with Lewis lung carcinoma cells and treated with cisplatin and/or QYG to evaluate the effect of QYG on cisplatin-induced tumoricidal activity. As shown in Fig. 6, QYG did not attenuate the tumoricidal effect of cisplatin, but rather lengthened the survival time of the xenograft model mice.

The results described above demonstrate that multiple pathways are responsible for tubular cell apoptosis during cisplatin-induced nephrotoxicity. Simultaneous inhibition of the various signaling pathways involved in cisplatin-induced nephrotoxicity may be necessary for the production of a therapeutic effect by a drug or TCM formulation. Although many *in vivo* and *in vitro* studies have been performed to evaluate measures intended to reduce cisplatin-induced nephrotoxicity, the reported protective effects of such procedures have been partial, highlighting the need to seek alternative strategies with greater efficacy and fewer toxic effects^48^,^49^,^50^. In this study, we showed for the first time that the TCM formula QYG exhibited significant therapeutic benefits against cisplatin-induced renal injury without reducing the antitumor activity of cisplatin. The renoprotective effects of QYG were mediated through multiple mechanisms of action, including inhibition of inflammation, suppression of apoptosis, reduction of platinum accumulation, and promotion of tubular cell regeneration. Because cisplatin must cross the plasma membrane of renal cells before interacting with intracellular targets to induce nephrotoxicity, we proposed that QYG protects tubular cells against cisplatin toxicity in a sequential manner, initially through regulation of cisplatin accumulation, followed by inflammation, and finally apoptosis. Because ginseng, the major plant constituent of QYG, possesses potent anti-inflammatory activities, modulation of inflammatory responses may also be a major contributor to the protective effect of QYG against cisplatin toxicity^51^. While the exact mechanisms and active compounds responsible for the nephroprotective effects of QYG are still unknown, our results demonstrate the need for further experiments to establish an empirical foundation for the use of QYG as an adjunct therapy with cisplatin in the clinic.

## Additional Information

**How to cite this article**: Teng, Z.-Y. *et al.* Ancient Chinese Formula Qiong-Yu-Gao Protects Against Cisplatin-Induced Nephrotoxicity Without Reducing Anti-tumor Activity. *Sci. Rep.* **5**, 15592; doi: 10.1038/srep15592 (2015).

## Supplementary Material

Supplementary Information

## Figures and Tables

**Table 1:** The identified phytochemical compounds and their concentrations in QYG extract detected by LC-MS.

No.	Identification	t_R_(min)	Formula	[M-H]–			Fragment ions	Contents(Mean ± SD; μg/g)
				Mean measuredmass (Da)	Theoretical exactmass (Da)	Mass accuracy(ppm)		
1	Rehmannioside D	2.21	C_27_H_42_O_20_	685.2191	685.2191	0.0	731, 505, 341, 179	–
*2	Melittoside	2.27	C_21_H_32_O_15_	523.1664	523.1663	0.2	569, 361, 342	605.35 ± 11.26
*3	Echinacoside	4.43	C_35_H_46_O_20_	785.2509	785.2504	0.6	623, 161	39.85 ± 5.92
4	Rehmapicrogenin	4.65	C_10_H_16_O_3_	183.1029	183.1021	4.4	139	−
5	Jionoside A1/A2	5.08	C_36_H_48_O_20_	799.2657	799.2661	–0.5	785, 623, 605, 477, 175	−
6	Rehmaionoside A/B	5.57	C_19_H_34_O_8_	389.2184	389.2175	2.3	435	−
*7	Acteoside	5.75	C_29_H_36_O_15_	623.1976	623.1976	0.0	461, 315, 161	43.55 ± 3.70
8	Jionoside B1/B2	6.11	C_37_H_50_O_20_	813.2817	813.2817	0.0	637, 619, 193, 175	−
*9	Isoacteoside	6.18	C_29_H_36_O_15_	623.1975	623.1976	–0.2	461, 315, 161	39.10 ± 0.40
10	Martynoside	8.57	C_31_H_40_O_15_	651.2285	651.2289	–0.6	475, 193, 175	−
*11	Rg_1_	8.88	C_42_H_72_O_14_	799.4845	799.4844	0.1	845, 637, 475	25.09 ± 0.16
*12	Re	8.94	C_48_H_82_O_18_	945.5425	945.5423	0.2	991, 799, 637, 475	28.01 ± 5.08
*13	Rf	12.30	C_42_H_72_O_14_	799.4828	799.4844	−2.0	845, 637, 475	67.58 ± 6.52
*14	Notoginsenoside R_2_	12.87	C_41_H_70_O_13_	769.4734	769.4738	−0.5	815, 637, 475	6.11 ± 0.08
*15	S-Rh_1_	13.49	C_36_H_62_O_9_	637.4293	637.4316	−2.6	683, 475	95.88 ± 3.47
*16	R-Rh_1_	13.94	C_36_H_62_O_9_	637.4324	637.4316	1.3	683, 475	79.72 ± 2.65
*17	Ro	14.54	C_48_H_76_O_19_	955.4894	955.4903	−0.9	793, 731, 569	135.17 ± 27.65
18	Rh_4_	20.51	C_36_H_60_O_8_	619.4202	619.4210	−1.3	665	−
19	Zingibroside R_1_	21.06	C_42_H_66_O_14_	793.4351	793.4374	−2.9	631, 613, 569	−
*20	20(S)-Rg_3_	21.65	C_42_H_72_O_13_	783.4884	783.4895	−1.4	829, 621, 459	63.17 ± 0.96
*21	20(R)-Rg_3_	21.79	C_42_H_72_O_13_	783.4882	783.4895	−1.7	829, 621, 459	18.31 ± 0.19
