# Supplementary material for: Ancient Chinese Formula Qiong-Yu-Gao Protects Against Cisplatin-Induced Nephrotoxicity Without Reducing Anti-tumor Activity
Source: Sci Rep. 2015 Oct 29;5:15592. doi: 10.1038/srep15592 (PMC4625150; doi:10.1038/srep15592)
Supplement: Supplementary Information [file srep15592-s1.pdf]

Ancient Chinese Formula Qiong-Yu-Gao Protects Against Cisplatin-Induced Nephrotoxicity Without Reducing Anti-tumor Activity

Zhi-Ying Teng[a,b†], Xiao-Lan Cheng[a,b†], Xue-Ting Cai[a,b], Yang Yang[a,b], Xiao-Yan Sun[a,b], Jin-Di Xu[c], Wu-Guang Lu[a,b], Jiao Chen[a,b], Chun-Ping Hu[a,b], Qian Zhou[a,b], Xiao-Ning Wang[b], Song-Lin Li[c*], Peng Cao[a,b*]

[a]Jiangsu Key Laboratory for Pharmacology and Safety Evaluation of Chinese Materia Medica, Nanjing University of Chinese Medicine, Nanjing 210023, China

[b]Laboratory of Cellular and Molecular Biology, Jiangsu Province Academy of Traditional Chinese Medicine, Nanjing 210028, China

[c]Department of Pharmaceutical Analysis and Metabolomics, Jiangsu Province Academy of Traditional Chinese Medicine, Nanjing 210028, PR China

* Corresponding author: Laboratory of Cellular and Molecular Biology, Jiangsu Province Academy of Traditional Chinese Medicine, 100 Shizi Street, Hongshan Road, Nanjing 210028, China. Tel.: +86 25 8560 8666; Fax: +86 25 8560 8666.

E-mail addresses: pcao79@yahoo.com (P. Cao), songlinli64@126.com (S.-L. Li).

[†]The authors contributed equally to this work.

Figure S1. The chemical structures of 21 constituents identified in the QYG extract.

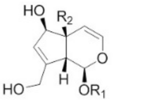

Compound	R_1	R_2
Rehmannioside D **(1)**	-Glc	-Glc^{2}-^{1}Glc
Melittoside **(2)**	-O-Glc	-Glc

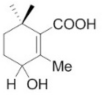

Rehmapicrogenin **(4)**

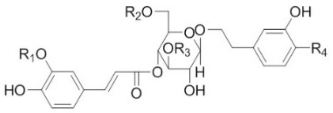

Compound	R_1	R_2	R_3	R_4
Echinacoside **(3)**	-H	-Glc	-Rha	-OH
Jionoside A1 **(5)**	-CH3	-Glc	-Rha	-OH
Acteoside **(7)**	-H	-H	-Glc	-OH
Jionoside B1 **(8)**	$-CH_3$	-Gal	-Rha	$-OCH_3$
Martynoside **(10)**	$-CH_3$	-H	-Rha	$-OCH_3$

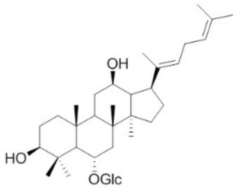

Rh_4 **(18)**

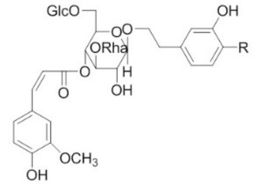

Compound	R
Jionoside A2 **(5)**	-OH
Jionoside B2 **(8)**	$-OCH_3$

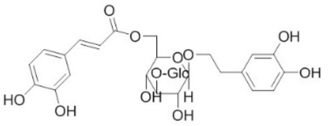

Isoacteoside **(9)**

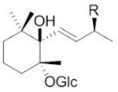

Compound	R
Rehmaionoside A **(6)**	-OH
Rehmaionoside B **(6)**	-H

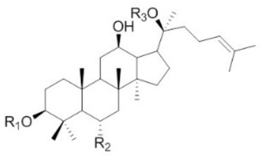

Ginsenoside	R_1	R_2	R_3
Rg_1 **(11)**	-H	-O-Glc	-Glc
Re **(12)**	-H	-O-Glc^{2}-^{1}Rha	-Glc
Rf **(13)**	-H	-O-Glc^{2}-^{1}Glc	-H
Notoginsenoside R_2 **(14)**	-H	-O-Glc^{2}-^{1}Xyl	-H
20S-Rh_1 **(15)**	-H	-O-Glc	-H
20S-Rg_3 **(20)**	-Glc2-1Glc	-H	-H

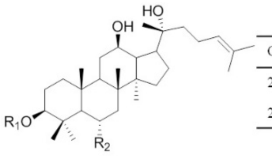

Ginsenoside	R_1	R_2
20R-Rh_1 **(16)**	-H	-O-Glc
20(R)-Rg_3 **(20)**	-Glc^{2}-^{1}Glc	-H

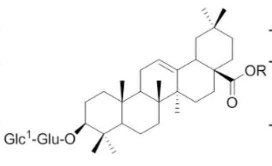

Ginsenoside	R
Ro **(17)**	-Glc
Zingibroside R1 **(19)**	-H
